# A Facile Electrochemical Sensor Based on PyTS–CNTs for Simultaneous Determination of Cadmium and Lead Ions

**DOI:** 10.3390/s18051567

**Published:** 2018-05-15

**Authors:** Ruyuan Jiang, Niantao Liu, Sanshuang Gao, Xamxikamar Mamat, Yuhong Su, Thomas Wagberg, Yongtao Li, Xun Hu, Guangzhi Hu

**Affiliations:** 1 Key Laboratory of Oil/Gas Fine Chemical Engineering, Ministry of Education and Autonomous Region, College of Chemistry and Chemical Engineering, Xinjiang University, Urumqi 830046, China; 18299443683@163.com (R.J.); liuninatao@163.com (N.L.); 2 Key Laboratory of Chemistry of Plant Resources in Arid Regions, State Key Laboratory Basis of Xinjiang Indigenous Medicinal Plants Resource Utilization, The Xinjiang Technical Institute of Physics and Chemistry, Chinese Academy of Sciences, Urumqi 830011, China; Sanshuang2018@163.com (S.G.); yongtao@ms.xjb.ac.cn (Y.L.); 3 Department of Physics, Umeå University, Umeå 90187, Sweden; thomas.wagberg@umu.se; 4 School of Material Science and Engineering, University of Jinan, Jinan 250022, China; mse_hux@ujn.edu.cn

**Keywords:** carbon nanotubes, sodium pyrene-1,3,6,8-tetrasulfonate, DPASV, simultaneous determination, Cd^2+^, Pb^2+^

## Abstract

A simple and easy method was implemented for the contemporary detection of cadmium (Cd^2+^) and lead (Pb^2+^) ions using 1,3,6,8-pyrenetetrasulfonic acid sodium salt-functionalized carbon nanotubes nanocomposites (PyTS–CNTs). The morphology and composition of the obtained PyTS–CNTs were characterized using scanning electron microscopy (SEM), energy dispersive spectrometry (EDS), and X-ray photoelectron spectroscopy (XPS). The experimental results confirmed that the fabricated PyTS–CNTs exhibited good selectivity and sensitivity for metal ion-sensing owing to the insertion of sulfonic acid groups. For Cd^2+^ and Pb^2+^, some of the electrochemical sensing parameters were evaluated by varying data such as the PyTS–CNT quantity loaded on the pyrolytic graphite electrode (PGE), pH of the acetate buffer, deposition time, and deposition potential. These parameters were optimized with differential pulse anodic sweeping voltammetry (DPASV). Under the optimal condition, the stripping peak current of the PyTS–CNTs/Nafion/PGE varies linearly with the heavy metal ion concentration, ranging from 1.0 μg L^−1^ to 90 μg L^−1^ for Cd^2+^ and from 1.0 μg L^−1^ to 110 μg L^−1^ for Pb^2+^. The limits of detection were estimated to be approximately 0.8 μg L^−1^ for Cd^2+^ and 0.02 μg L^−1^ for Pb^2+^. The proposed PyTS–CNTs/Nafion/PGE can be used as a rapid, simple, and controllable electrochemical sensor for the determination of toxic Cd^2+^ and Pb^2+^.

## 1. Introduction

Toxic heavy metals are one of the most serious threats to the environment and have drawn more attention because of their non-biodegradable and persistent nature [1,2,3]. Many industries, such as those for battery outputs, metal plating apparatus, mining activities, electroplating, fossil combustibles, pesticides and paper, tanneries, manure, diverse plastics, and metallurgies, are major sources of the toxic heavy metals [4,5]. In the past decade, the rapid increase in industrial production has resulted numerous heavy metals invading the ecosystem [6,7]. These heavy metal ions, such as Cd^2+^ and Pb^2+^, possess the slow process characteristic of toxicity fading and are difficult to degrade by microorganisms; they are typically considered to be highly toxic and hazardous to human health and the natural environment even at trace levels [8,9]. It is therefore necessary to develop fast, economic, and timely methods for monitoring Cd^2+^ and Pb^2+^ concentrations at trace levels [10,11].

Currently, atomic absorption spectroscopy (AAS), atomic fluorescence spectrometry (AFS), atomic emission spectroscopy (AES), inductively coupled plasma mass spectrometry (ICP-MS), and coupled plasma optical emission spectrometer (ICP-OES) have primarily been used for monitoring toxic heavy metals in contaminated water [12,13,14]. However, although most of these typical methods have good accuracy and high sensitivity, they still have some disadvantages in that they are time-consuming, expensive, and complicated pretreatment procedures. Moreover, these techniques typically generate waste-containing organic solvents and/or gas during the measurement, thereby rendering the traditional quantification methods more complicated and expensive [15]. Owing to its simplicity, cost-effectiveness, and high sensitivity, heavy metal quantification methods based on electrochemical techniques have been widely used for detecting toxic metal ions, such as Cd^2+^ and Pb^2+^ [16]. The chemical groups and physical structure of the electrode surface are critical in the electrode reaction process [17]. Owing to the rapid development of chemically modified electrodes, various kinds of functionalized materials have been applied to modify the electrode surface for improving the electrochemical performance in both electroanalysis and electrocatalysis [18,19].

Differential pulse anodic stripping voltammetry (DPASV) is one of the most important electrochemical techniques, which can make trace analysis of heavy metal ions accurate by electrochemically depositing the heavy metal onto the working electrode surface [20]. Its remarkable improvement in sensitivity is attributed to the combination of the effective preconcentration step and the following measurement procedures [21]. Herein, the DPASV current signal strongly depends on the electrode modification material [22].

Because of its large surface area, excellent conductivity, and chemical inertness, carbon-based nanomaterials, such as carbon nanotubes (CNTs) and graphene, have been regarded as good candidates for functionalizing the electrode surface, showing a lower detection limit and a wider linear range towards the target analytes [23]. Carbon nanotubes have been given the most consideration by scientists owing to their special texture, excellent electron conductivity, and physicochemical characteristics [16,24,25]. The graphitic hollow nanotexture of CNTs with particularly high surface area makes them highly adsorbent towards toxic metal contaminants [26]. CNTs with large surface area and good porosity have strong interactions with heavy metal ions during the wastewater purification treatment in industrial manufacturing processes [27]. However, amorphous carbon on the CNT surface is hydrophobic, and significantly affects the adsorption ability of CNTs towards toxic heavy metal ions. To improve the accumulation performance of CNT electrodes, a variety of strategies for modifying the CNT surface have been explored [28]. As a kind of organic salt containing –SO_3_H, 1,3,6,8-pyrenetetrasulfonic acid sodium salt (PyTS) is readily available, inexpensive, and extremely water-soluble. CNTs can be easily functionalized by PyTS through a strong π–π stacking interaction between PyTS and CNTs [29]. After being functionalized with PyTS, CNTs perform excellent dispersity in water because of the subsistence of the negatively charged sulfonic group (–SO_3_^2−^) on the CNT surface [30].

Here, we report an efficient electrochemical method for detecting Cd^2+^ and Pb^2+^ based on PyTS-functionalized CNTs. PyTS–CNTs were synthesized using a facile ultrasonic method and subsequently used to prepare PyTS–CNT-modified electrodes for monitoring Cd^2+^ and Pb^2+^ in environmental samples. This proposed protocol is a simple, fast, and controllable technique to detect toxic heavy metal ions.

## 2. Experimental

### 2.1. Reagents and Materials

The experimental reagents were analytical grade and used as received without any further purification. PyTS (Haite Plastic Pigment Co., Ltd., Kunshan, China) and CNTs (Cangji Nanometer Technology Co., Ltd., Nanjing, China) were purchased for preparing the PyTS–CNT nanocomposite. Nafion solution (5 wt %, Sigma-Aldrich, Shanghai, China) and 2-propanol (Titan Scientific Co., Ltd., Shanghai, China) were used for preparing the chemically modified electrode. Standard cadmium solution (Cd^2+^, 1000 μg mL^−1^) and standard lead solution (Pb^2+^, 1000 μg mL^−1^) were supplied by Alfa Aesar (Shanghai, China). For the pH adjustment, a series of HAc–NaAc buffers containing acetic acid (Beilian Chemical Co., Ltd., Tianjin, China) and sodium acetate (Tianjin Baishi Chemical Co., Ltd.) at a concentration of 0.1 mol L^−1^ were prepared by mixing the two salts at different ratios. All the test solutions were prepared with deionized water (≥18.2 Ω). All the measurements were obtained at room temperature (18–25 °C).

### 2.2. Instruments

All electrochemical measurements were recorded on the CHI1040C electrochemical workstation (CHI Instrument, Shanghai, China) with a three-electrode setup consisting of a pyrolytic graphite electrode (d = 3 mm, 0.07 cm^2^ geometric area) as the working electrode, a Pt wire as the counter electrode (counter electrode), and a silver/silver chloride electrode as the reference electrode (Ag/AgCl). The solution pH was measured using a digital pH meter (Mettler-Toledo, Shanghai, China). The morphology of PyTS–CNTs was characterized using scanning electron microscopy (SEM, Carl Zeiss, Germany) in conjunction with energy dispersive spectroscopy (EDS, BRUKER, Germany). X-ray photoelectron spectroscopy (XPS) analysis was performed on the Thermo Scientific ESCALAB 250XI X-ray photoelectron spectrometer.

### 2.3. Preparation of PyTS–CNTs

**Scheme 1:**
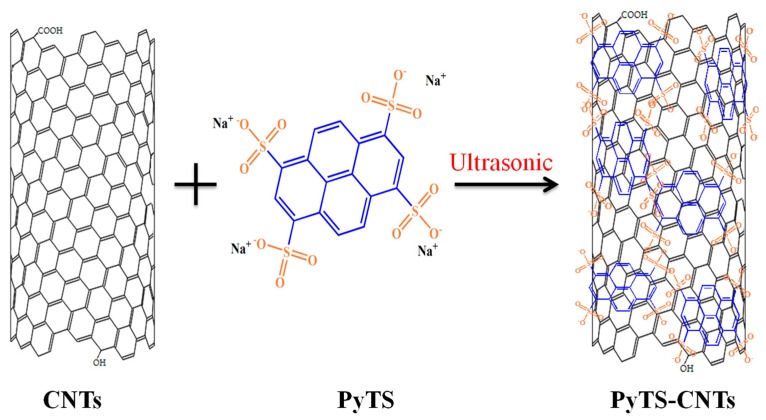
Schematic diagram of the fabrication procedure of 1,3,6,8-pyrenetetrasulfonic acid sodium salt-functionalized carbon nanotubes (PyTS–CNTs).

PyTS–CNTs were prepared by sonicating the mixture of PyTS and CNTs and then centrifuging the mixture suspension. The CNTs (4 mg) and PyTS (80 mg) were dispersed separately in deionized water and then mixed together [31]. The mixture solution was ultrasonicated, followed by vigorous stirring at room temperature for 2 h. Finally, the well-dispersed CNT suspension was centrifuged and rinsed with distilled water twice. The preparation of the PyTS–CNTs is illustrated in Scheme 1.

### 2.4. Preparation of PyTS–CNT-Modified PGE

PyTS–CNTs were redispersed into a mixture of deionized water and 2-propanol (1:4, *v*:*v*) containing 0.1 wt % Nafion as a binder to form a 2 mg mL^−1^ slurry. The slurry was then sonicated for 30 min. Prior to electrode modification, the pyrolytic graphitic electrode (PGE, Ф = 3 mm) was carefully polished with alumina slurry (0.05, 0.3, and 1.0 μm in particle size), and cleaned with ethanol and distilled water, sequentially. The pretreated PGE was dried by flowing N_2_. The PyTS–CNTs/Nafion/PGE was prepared by casting 4 μL of homogeneous suspensions onto the PGE surface and then dried at room temperature. 

### 2.5. DPASV Analysis

Cyclic voltammetry (CV) and DPASV measurements were performed on the CHI 1040C electrochemical workstation in 0.1 M of fresh HAc–NaAc (pH = 5.0) solution containing a certain concentration of the target heavy metal ions (Cd^2+^ and Pb^2+^). The target metals ions were reduced and then electrodeposited on the surface of the PyTS–CNTs/Nafion/PCE at a negative potential of −1.2 V for 270 s under stirring. After an equilibration of 10 s, the DPASV measurements were performed in a potential window ranging from −0.6 V to −1.0 V with an amplitude of 50 mV, pulsation of 50 ms, time interval of 0.5 s, and a potential step accretion of 4 mV. A “cleaning” step was performed by maintaining the conditioning potential (*E*_cond_) at 0.6 V for 60 s after each DPASV measurement to remove the residual heavy metals on the PyTS–CNT hybrid film.

## 3. Results and Discussion

### 3.1. PyTS–CNT Characterization

**Figure 1:**
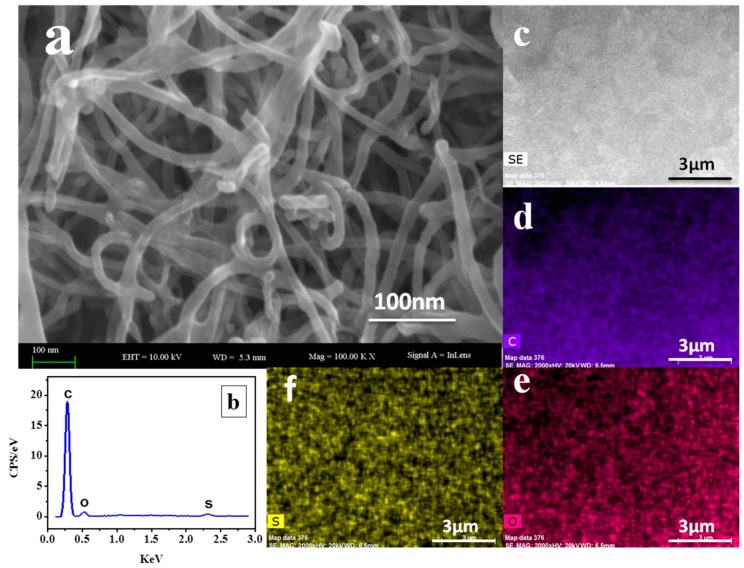
(**a**) SEM images of the synthesized PyTS–CNTs, (**b**) EDS analysis of PyTS–CNTs, and the related EDS mapping of carbon (**d**), oxygen (**e**), and sulfur (**f**) in the selected area (**c**).

The structural and elementary distribution of the PyTS–CNTs were characterized using SEM and EDS mapping. Their surface morphology was investigated using SEM. As shown in Figure 1a, the abundant PyTS–CNTs interweaved to form a netlike structure. The PyTS–CNT nanocomposites show better membrane-forming ability compared to pure CNTs, which show weak electron conductivity and more disconnections. The EDS spectrum of the PyTS–CNTs, as shown in Figure 1b, confirms the existence of sulfur element within the PyTS–CNT nanocomposites. In addition, the selected SEM image in Figure 1c and the corresponding EDS mappings (Figure 1d–f) indicate that the primary elements (C, O, and S) were uniformly distributed on the PyTS–CNTs surface. The S element mapping confirms that sulfur-containing groups were successfully functionalized onto the CNT surface.

**Figure 2:**
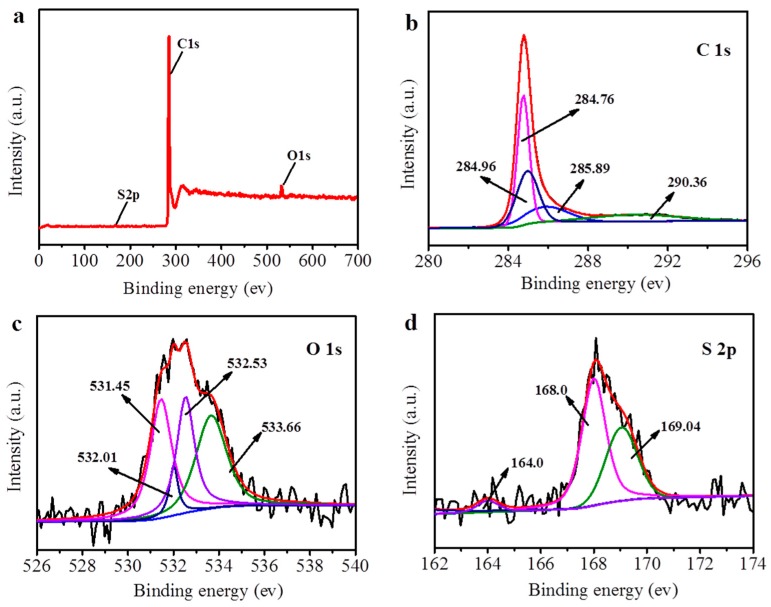
Wide-scan XPS spectra (**a**) and high-resolution XPS spectra of C1s (**b**), O1s (**c**), and S2p (**d**) of PyTS–CNTs.

The chemical valence of the elements on the surface of the CNTs can be identified by XPS spectra. As shown in Figure 2a, the contents of carbon, oxygen, and sulfur on the surface of the PyTS–CNTs have an atom % of 96.85%, 2.8%, and 0.35%, respectively. The wide-scan XPS survey of the PyTS–CNTs exhibits two powerful curves representing graphitic C1s (284.79 eV) and O1s (532.14 eV), and a weak S2p peak around 168.16 eV. The high-resolution C1s spectrum consists of four deconvoluted peaks, as shown in Figure 2b, which can be ascribed to the C–C (284.76 eV), C–S [32] (284.96 eV), C–O (285.89 eV), and O–C=O (290.36 eV) groups, from left to right [33]. The high-resolution XPS spectra of O1s (Figure 2c) consist of C=O (532.01 eV), O–H (532.53 eV), C–O (533.0 eV), and O–S (533.66 eV) groups. Furthermore, to further ascertain the bonding configurations of S atoms, the high-resolution S2p spectrum was analyzed, as shown in Figure 2d. The small peak of S2p at 164.0 eV can be ascribed to the spin–orbit coupling of the C–S bond in PyTS, while the doublet peak at 168.1 eV and 169.3 eV are characteristic of the S–O bond [34]. Notably, the PyTS peak is the most prominent in the spectrum of the PyTS–CNT material, which indicates that PyTS has been successfully decorated onto the CNT surface.

### 3.2. Electrochemical Behaviors of Various Modified Electrodes

**Figure 3:**
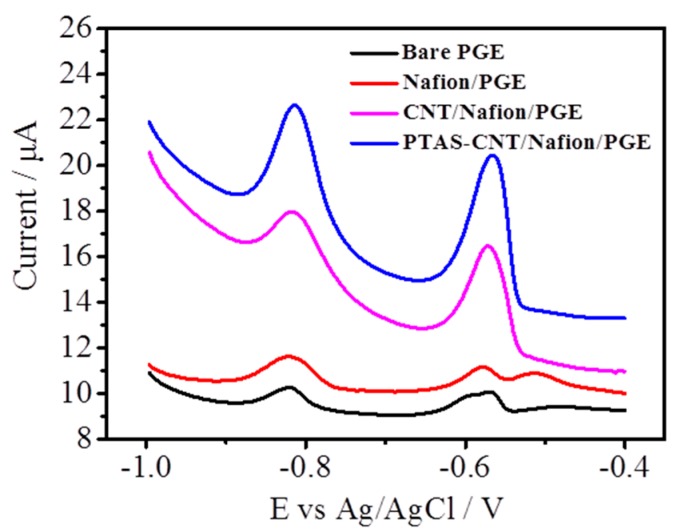
Differential pulse anodic sweeping voltammetry (DPASV) curves of 50 μg L^−1^ Cd^2+^ and Pb^2+^ on bare pyrolytic graphite electrode (PGE) (black), Nafion/PGE (red), CNTs/Nafion/PGE (pink), and PyTS–CNTs/Nafion/PGE (blue) in 0.1 mol L^−1^ HAc–NaAc (pH = 5.0) with Nafion concentration: 0.1 wt %; deposition potential: −1.2 V; deposition time: 270 s; amplitude: 50 mV; pulse width: 50 ms; potential step: 4 mV.

To investigate their electrochemical response towards heavy metal ions, the results for different modified electrodes, including bare PGE, Nafion/PGE, CNTs/Nafion/PGE, and PyTS–CNTs/Nafion/PGE, were carefully compared. As shown in Figure 3, stripping peaks appear for both Cd^2+^ and Pb^2+^ for these three electrodes. The weakest stripping peaks were observed at bare PGE in the voltage range from −1.0 V to −0.4 V (vs. Ag/AgCl) after accumulating at −1.2 V for 270 s. The stripping peak currents were 0.24 µA for Cd^2+^ and 0.55 µA for Pb^2+^.

Slightly increased stripping peak currents were observed for Nafion/PGE, with the peak current of 1.29 µA for Cd^2+^ and 0.70 µA for Pb^2+^. Meanwhile, the stripping peak currents of both Cd^2+^ and Pb^2+^ are higher than those of bare and Nafion/PGE. Two well-defined stripping peaks were obtained with the CNTs/Nafion/PGE (5.39 µA for Cd^2+^ and 5.48 µA for Pb^2+^) and PyTS–CNTs/Nafion/PGE (7.27 µA for Cd^2+^ and 6.43 µA for Pb^2+^). Compared with bare PGE, Nafion/PGE, and CNTs/Nafion/PGE, the PyTS–CNTs/Nafion/PGE shows better sensing performance towards heavy metal ions, which can be attributed to the following: (1) The oxygen-containing groups, such as C=O, –OH, –COOH, and –SO_3_^2−^, are critical for heavy metal accumulation; (2) the PyTS–CNTs provide excellent affinity for Cd^2+^ and Pb^2+^, and the interlaced PyTS–CNTs provide a three-dimensional network structure, which is beneficial for the heavy metal ion diffusion. The results above indicate that PyTS–CNTs/Nafion/PGE has the best sensing properties for the simultaneous determination of Cd^2+^ and Pb^2+^, with a peak potential separation of 340 mV.

### 3.3. Optimization of Experimental Conditions

Some stripping parameters affected the peak currents of Cd^2+^ and Pb^2+^ during the DPASV measurements. Batch studies were performed to evaluate the effects of various tests parameters, such as the mass loading of PyTS–CNTs onto the PGE, deposition time, deposition voltage, and acetate buffer pH. 

**Figure 4:**
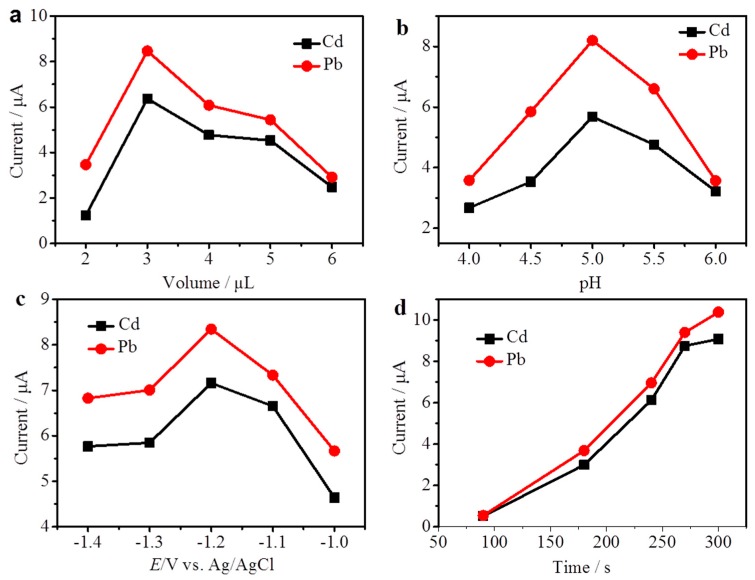
Effects of the measurement conditions: (**a**) quantity of PyTS–CNTs loaded onto PGE, (**b**) solution pH value, (**c**) deposition potential, (**d**) deposition time. The other parameters are equal to those in Figure 3.

The thickness of the PyTS–CNT membrane also affects the stripping peak currents of Cd^2+^ and Pb^2+^. The thickness was defined by the mass loading of the PyTS–CNT slurry. Figure 4a shows the mass loading effect of the PyTS–CNT slurry towards the stripping peak currents of Cd^2+^ and Pb^2+^. For Cd^2+^ and Pb^2+^, the maximum stripping peak currents were obtained when 3 µL of slurry was cast onto the PGE. This phenomenon was likely caused by the increasing PyTS–CNTs that introduced more active sites on the electrode surface, which can improve the electron transportation and accumulate more heavy metal ions at the PyTS–CNTs/PGE. However, excessively increasing the volume of the PyTS–CNT slurry will result in a thicker film, which slows down mass transport and prevents the diffusion of the target analytes at the modified electrode. Therefore, 3 µL of PyTS–CNTs slurry was chosen as the optimal mass loading volume.

The effect of solution acidity (pH), a significant factor in the DPASV process, was also carefully investigated, because the solution pH affects sorption behavior of Cd^2+^ and Pb^2+^. As shown in Figure 4b, the electrochemical responses of both Cd^2+^ and Pb^2+^ increased when the solution pH increased from 4.0 to 5.0. This is likely due to the competitive adsorption between the positive protons and target ions. When the pH is above 5.0, the stripping peak currents of Cd^2+^ and Pb^2+^ decreased rapidly, owing to the formation of heavy metal hydroxides in a weak acid solution; therefore, the pH of 5.0 was selected as the optimized solution acidity for future measurements. 

Figure 4c shows the effect of deposition potential on the stripping peak currents at a potential window ranging from −1.0 V to −1.4 V. When the deposition voltage negatively shifted from −1.0 V to −1.2 V, an obvious increase in the stripping peak current occurred for both Cd^2+^ and Pb^2+^. While the accumulation potential was lower than −1.2 V, the stripping peak currents were dramatically reduced because of the serious effect of the hydrogen evolution reaction (HER). Therefore, the optimal deposition potential was selected as −1.2 V for both Cd^2+^ and Pb^2+^ detections. 

Figure 4d shows the effect of deposition time, from 90 s to 300 s. The stripping currents of Cd^2+^ and Pb^2+^ increased from 90 s to 270 s, indicating that a longer deposition time caused more Cd^2+^ and Pb^2+^ to be deposited onto the sensing electrode surface to form alloys. However, the peak currents of Cd^2+^ and Pb^2+^ increased slowly when the deposition time was above 270 s. This can be attributed to the adsorption saturation of the active site on the modified electrode. Therefore, 270 s was selected as the optimal deposition time.

### 3.4. Analyses for Determination of Cd(II) and Pb(II)

**Figure 5:**
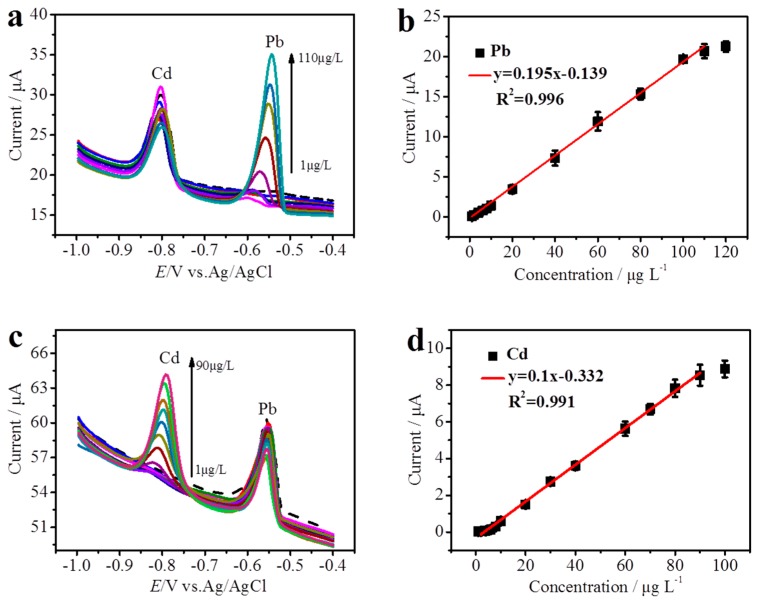
DPASV curves of different Pb^2+^ concentrations (**a**) and the corresponding calibration plot (**b**) of anodic peak current as a function of concentration in the presence of 50 µg L^−1^ Cd^2+^; DPASV curves of different Pb^2+^ concentrations (**c**) and the corresponding calibration plot (**d**) of anodic peak current as a function of concentration in the presence of 50 µg L^−1^ Pb^2+^. The other parameters are equal to those in Figure 3.

The PyTS–CNTs/Nafion/PGE was applied to determine the concentration of Cd^2+^ and Pb^2+^ with the DPASV method under the optimized conditions. The investigation was performed by maintaining one species’ concentration constant and changing the other heavy metal ion’s concentration. As shown in Figure 5a,c, two separated stripping peaks were observed at approximately −0.82 V and −0.55 V, attributed to the stripping process of Cd^2+^ and Pb^2+^, respectively. Figure 5a,b show that the stripping current is linear with the Pb^2+^ concentration, ranging from 1 µg L^−1^ to 110 µg L^−1^, for a constant Cd^2+^ concentration of 50 µg L^−1^. The linear regression equation was obtained as y = 0.195x − 0.139, (R^2^ = 0.996, y: current μA, x: concentration µg L^−1^). This indicates that the Cd^2+^ concentration had an excellent linear correlation with the stripping current, while that of its counterpart, Pb^2+^, remained unchanged. Similar results were also observed, as shown in Figure 5c,d; the stripping peak current of Cd^2+^ is also linear with its concentration, ranging from 1 µg L^−1^ to 90 µg L^−1^, in the presence of 50 µg L^−1^ of Pd^2+^. The correlation equation can be defined by y = 0.10x + 0.332 (R^2^ = 0.991, y: current μA, x: concentration µg L^−1^). These results indicate that the obtained PyTS–CNT-modified electrode can be used for the simultaneous determination of Cd^2+^ and Pb^2+^. The limits of detection were estimated to be 0.8 µg L^−1^ for Cd^2+^ and 0.02 µg L^−1^ for Pb^2+^ (S/N = 3), which are approximately 6 and 500 times lower than the World Health Organization standard for drinking water. 

The results of some previous studies were compared with those of the proposed electrochemical sensor electrode in terms of the sensing performance, including the limits of detection and linear ranges (Table 1). As shown, it can be seen that the detection limits of the fabricated PyTS–CNTs/Nafion/PGE sensor are lower than those in most previous reports using carbon nanotubes, graphene, and other core–shell nanoparticles. It is clear that the PyTS-functionalized CNTs possess a large surface area, fast electron transfer, and rich active sites for toxic metal ions. Additionally, the PyTS–CNTs/Nafion/PGE shows an improved analytical performance towards heavy metal ions in terms of a wider linear range and low detection limits; hence, its potential applications are wider, and it can be used as an effective electrochemical sensing platform for the determination of toxic metal ions owing to its superior analytical productivity.

### 3.5. Interferences

**Figure 6:**
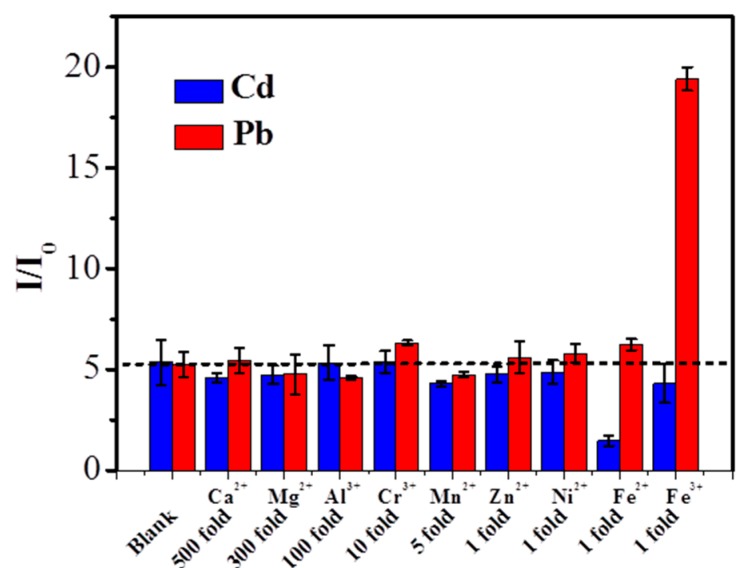
Interference on the stripping peak currents of 50 μg L^−1^ Cd^2+^ (blue) and 50 μg L^−1^ Pb^2+^ (red) at the PyTS–CNTs/Nafion/PGE. I_0_ and I represent the stripping current of 50 μg L^−1^ Cd^2+^ and 50 μg L^−1^ Pb^2+^ in the absence and presence of interfering ions, respectively. DPASV detection conditions are referred to in Figure 3.

The anti-interference effect of the proposed sensor was also evaluated by adding some common interfering ions into the mixture solution containing 50 μg L^−1^ Cd^2+^ and 50 μg L^−1^ Pb^2+^. By allowing a stripping current change of 10% for simultaneously detecting 50 μg L^−1^ Cd^2+^ and 50μg L^−1^ Pb^2+^, 500-fold Ca^2+^, 300-fold Mg^2+^, 100-fold Al^3+^, 10-fold Cr^3+^, 5-fold Mn^2+^, 1-fold Zn^2+^, and Ni^2+^ can be effectively tolerated. As shown, the significant amounts of NO_3_^2−^, SO_4_^2−^, and PO_4_^3−^ ions had a negligible effect on the stripping currents of Cd^2+^ and Pb^2+^ [45]. The results in Figure 6 indicate that these metal ions do not interfere with the detection of target heavy metal ions. However, high concentration of Co^2+^, Fe^2+^, and Fe^3+^ had a negative influence on the stripping peak currents of Cd^2+^ and Pb^2+^, owing to their competing adsorption of Cd^2+^ and Pb^2+^ on the PyTS–CNT electrode surface during the pre-accumulation step. We found that the interference of these ions is negligible on our proposed electrode by using a pretreatment process, such as a masking agent.

### 3.6. Repeatability and Reproducibility 

As shown in Table 2, the reproducibility and repeatability of the PyTS–CNTs were investigated by repetitive measurements of 50 μg L^−1^ Cd^2+^ and 50 μg L^−1^ Pb^2+^ solutions under the optimal conditions. Repeatability of the proposed electrochemical sensor was examined using one modified electrode for five tests, and the relative standard derivation (RSD) was 2.8% for Cd^2+^ and 2.4% for Pb^2+^ in five calculations. Meanwhile, the five fabricated electrodes exhibited similar electrochemical responses with RSDs of 6.4% for Cd^2+^ and 4.2% for Pb^2+^, indicating the satisfactory reproducibility of the proposed electrode. These results demonstrate the good repeatability and reproducibility of the PyTS–CNTs/PGE.

## 4. Conclusions

In summary, PyTS–CNTs were prepared using a one-pot and facile sonochemical synthesis method. The as-prepared PyTS–CNTs were used to prepare the PyTS–CNTs/Nafion/PGE electrochemical sensor to evaluate its sensing performance towards heavy metal ions of Cd^2+^ and Pb^2+^. Experimental results confirm that the PyTS–CNTs possess good electrochemical response and sensitivity to detect toxic metal ions. The fabrication of the PyTS–CNT electrode as an electrochemical sensor was fast, simple, and controllable. More importantly, the facile and environmentally friendly electrochemical sensor may provide a cost-effective platform for the green, facile, and sensitive analysis of Cd^2+^ and Pb^2+^ in environmental samples.

## Figures and Tables

**Table 1:** Comparison of analytical performance of various modified electrodes towards Cd^2+^ and Pb^2+^.

Electrode Substrate	Method	Deposition Time (s)	Linear Range (μg L^−1^)		Limit of Detection (µg L^−1^)		Reference
			Cd^2+^	Pb^2+^	Cd^2+^	Pb^2+^	
PA/PPy/GO/GCE	DPASV	200	5–150	5–150	2.13	0.41	[10]
G/Nafion/MWCNTs/Bi/GCE	DPASV	180	0.5–30	0.5–30	0.1	0.2	[19]
CNF/Nafion/GCE	OSWSV	300	-	-	0.19	0.31	[21]
N–CNT/GCE	SWASV		11–11,200	2–1450	5.6	0.21	[35]
SbNP^/^MWCNT/CPE	DPASV	120	10–60	10–60	0.8	0.7	[36]
Bi/Nafion/RGO-GNPs/GCE	SWASV	140	1–90	1–90	0.12	0.08	[37]
BiOCl/MWCNT–GCE	SWASV	120	5–50	5–50	1.2	0.6	[38]
SNAC/GCE	DPASV		10.1–539.6	18.6–1180.0	2.7	1.2	[39]
Bi/IPA-treated CTE	SWASV	300	5–110	5–110	1.08	0.87	[40]
							
MWCNTs/Bi–GCE	DPASV	180	0.5–30	0.5–30	0.1	0.2	[41]
Au–GN–Cys/GCE	SWASV	800	0.4–40	0.5–40	0.1	0.05	[42]
Bi/MGF–Nafion/GCE	DPASV	360	2–70	0.5–110	0.5	0.1	[43]
Diatomite–MPTMS/GCE	DPASV	300	20–300	20–150	15.9	6.9	[44]
PyTS–CNTs/Nafion/PGE	DPASV	270	1–90	1–100	0.8	0.02	This work

PA: phytic acid. PPy: polypyrrole. GO: graphene oxide. G: graphene. MWCNTs: multiwalled carbon nanotubes. SbNP: antimony nanoparticles. CPE: carbon paste electrode. BiOCl: bismuth-oxychloride. SNAC: spherical carbon nanoparticle decorated activated carbon IPA: isopropyl alcohol. CTE: carbon thread electrode. Au–GN–Cys: gold nanoparticle–graphene–cysteine composite. MGF: mesoporous graphene framework. MPTMS: 3-mercaptopropyltrimethoxysilane.

**Table 2:** Reproducibility and repeatability of the PyTS–CNTs towards 50 μg L^−1^ Cd^2+^ and Pb^2+^.

	Analyte	1	2	3	4	5	Found (μg L^−1^)	RSD (%)
Reproducibility *n* = 5	Cd^2+^	4.582	5.201	4.431	4.886	4.607	4.741 ± 0.304	6.4
	Pb^2+^	6.421	6.902	6.175	6.474	6.674	6.529 ± 0.274	4.2
Repeatability *n* = 1	Cd^2+^	4.713	4.469	4.734	4.5	4.717	4.627 ± 0.130	2.8
	Pb^2+^	6.83	6.831	6.805	6.458	6.807	6.746 ± 0.162	2.4
